# Supplementary material for: Plasma microRNA Signature as Companion Diagnostic for Abiraterone Acetate Treatment in Metastatic Castration-Resistant Prostate Cancer: A Pilot Study
Source: Int J Mol Sci. 2024 May 21;25(11):5573. doi: 10.3390/ijms25115573 (PMC11171781; doi:10.3390/ijms25115573)
Supplement: Supplementary file 1 [file ijms-25-05573-s001.zip › ijms-2962670-supplementary.pdf]

Table S1. Patients' and tumor's characteristics. Gleason score, enzalutamide treatment (ENZ), docetaxel treatment (DCX), cabazitaxel treatment (CBX), abiraterone acetate treatment period (AA duration), objective response to AA treatment (OR), PSA levels changes after AA treatment (PSA), progression free survival after AA treatment (PFS). AA = abiraterone acetate; MR = mixed response; NE = not evaluable; DP = disease progression; PR = partial response; S = PSA stabilization; SD = stable disease; UNK = unknown; <50% = PSA reduction less than 50% compared to baseline; >50% = PSA reduction more than 50% compared to baseline; Y=yes ; N=no. In bold, the patients used for the Exiqon panel analysis.

	Patient code	Age	Gleason Score	ENZ	DCX	CBX	AA Duration (months)	OR	PSA	PFS (months)
Non-responders	**P1**	75	8	Y	Y	Y	3.93	DP	<50%	3.9
	**P2**	84	7	Y	Y	N	2.93	NE	<50%	2.7
	**P3**	85	8	N	Y	N	4.03	DP	<50%	3.7
	**P4**	78	9	Y	Y	Y	6.8	MR	<50%	5.4
	**P5**	75	8	Y	Y	Y	3.77	DP	<50%	3.7
	**P6**	80	8	N	Y	N	3.73	DP	S	3.7
	**P7**	73	7	Y	Y	N	2.47	NE	<50%	2.4
	**P8**	78	7	N	Y	N	1.93	NE	<50%	1.9
	**P9**	77	8	Y	Y	N	3.97	DP	>50%	3.9
	P10	76	UNK	Y	Y	Y	3.87	DP	<50%	3.8
	P11	64	9	Y	Y	Y	4.8	DP	>50%	4.7
	P12	74	9	Y	Y	N	4.63	NE	<50%	4.2
	P13	78	7	N	Y	N	3.27	DP	<50%	3.7
	P14	68	8	N	Y	Y	5.57	MR	<50%	5.5
	P15	46	9	Y	Y	Y	4.63	MR	>50%	4.6
	P16	75	9	N	Y	N	13.1	DP	>50%	1.8
	P17	70	8	N	Y	N	4.7	DP	<50%	4.6
	P18	63	10	N	Y	N	3.73	DP	>50%	3.7
	P19	82	6	N	Y	N	3.93	DP	<50%	3.9
	P20	76	9	Y	Y	N	2.67	DP	<50%	3.9
	P21	70	10	N	Y	Y	5.6	DP	>50%	5.5
Responders	**P22**	70	9	N	Y	Y	12.17	PR	<50%	12
	**P23**	84	7	N	Y	N	4.67	PR	>50%	16.6
	**P24**	73	7	N	Y	Y	20.8	PR	>50%	20.8
	**P25**	81	7	N	Y	N	9.13	PR	<50%	9
	**P26**	75	7	N	Y	N	13.37	PR	<50%	13.2
	**P27**	74	9	N	Y	N	21.33	PR	>50%	21.7
	**P28**	76	6	Y	Y	Y	15.1	SD/PR	>50%	14.9
	**P29**	80	9	N	Y	N	9.33	PR	>50%	8.7
	**P30**	70	9	N	Y	Y	7.5	MR	S	7.8
	P31	72	UNK	N	Y	Y	14.3	PR	>50%	13.1
	P32	84	UNK	N	N	N	21.7	PR	>50%	21.7
	P33	73	9	Y	Y	Y	4.43	PR	>50%	14.8

Table S2. Fold change and literature review of miRNAs candidates.

miRNA	Fold Change (NR vs. R)	Literature Review
**miR-103a-3p**	1.1	[1–4]
**miR-144-3p**	-4.1	[5–8]
**miR-182-5p**	4.9	[9–11]
**miR-29b-3p**	-2.2	[12,13]
**miR-331-5p**	6.9	[14]
**miR-33a-5p**	-4.7	[15,16]
**miR-363-3p**	-2.4	[17–19]
**miR-378a-5p**	-4.7	[20–24]

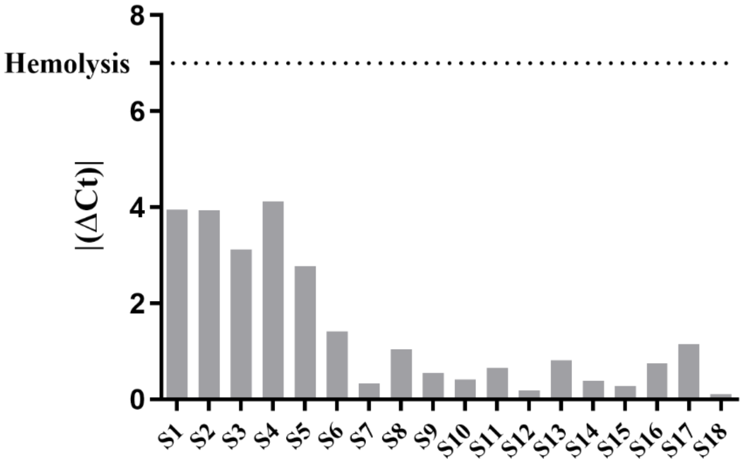

Figure S1. Hemolysis check for Exiqon miRNome panels data. The absolute difference among Ct values of miR-451a-5p and miR-23a-3p indicates that no hemolysis was present in the samples analyzed following the criteria of Blondal et al. ($\Delta Ct > 7$) [25].

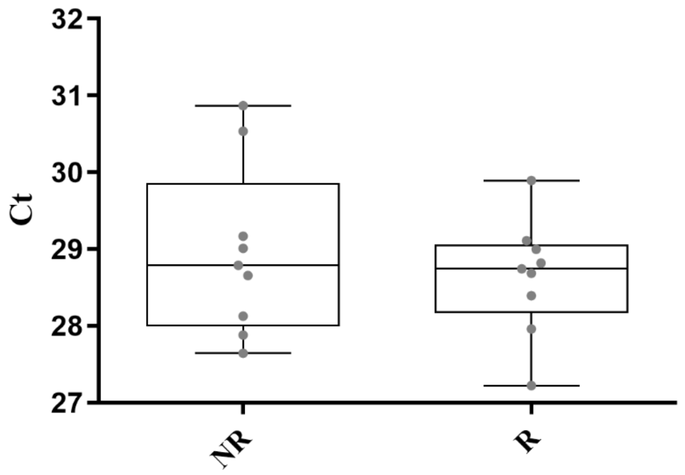

Figure S2. Ct values of miR-425-5p in candidate analysis, in non-responder (NR) and responder patients (R).

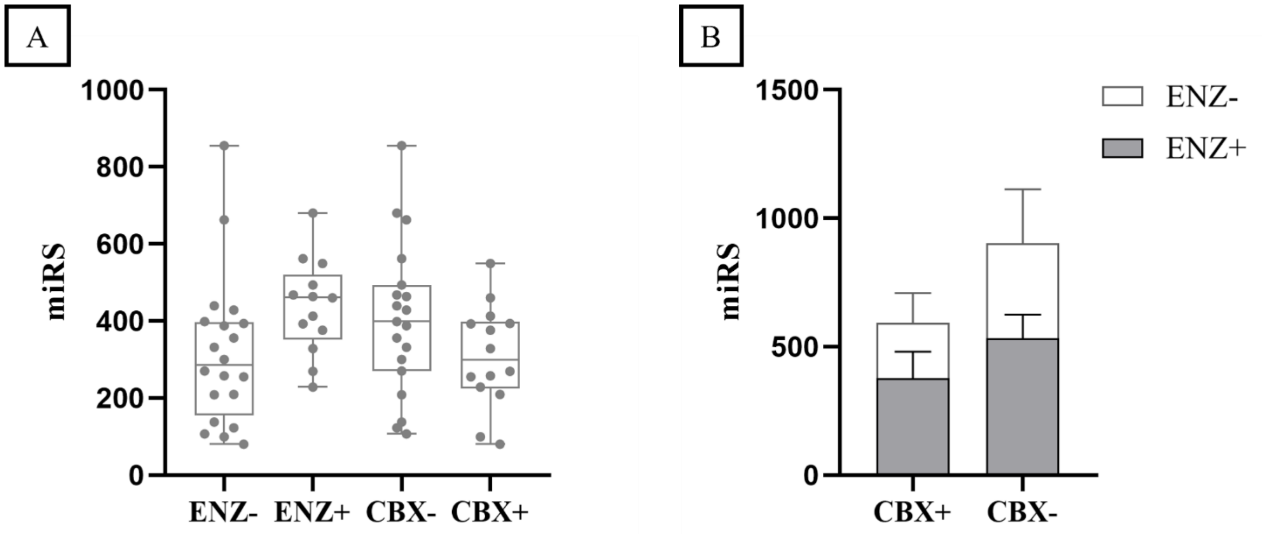

Figure S3. (**A**) Patients were grouped based on miRS data for enzalutamide and cabazitaxel treatment. The Student's T-test (ENZ- vs ENZ+ and CBX- vs CBX+) did not show significant differences. (**B**) Patients were also grouped based on miRS for combined treatment of enzalutamide and cabazitaxel. The 2-way ANOVA dit not show significant interactions.

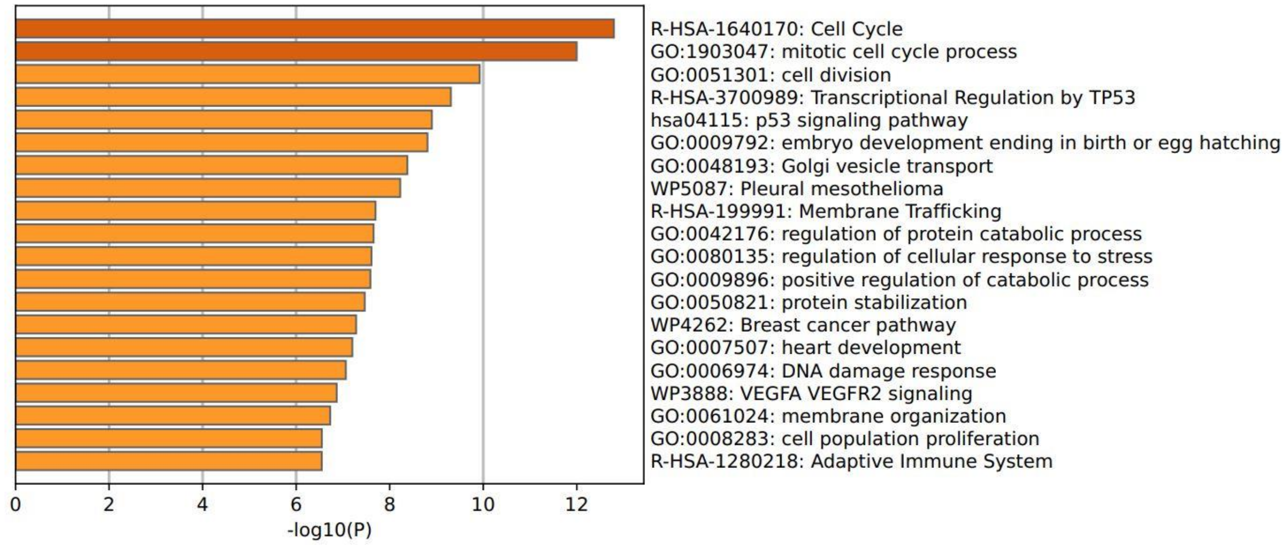

Figure S4. Gene ontology analysis (Metascape) on shared biological processes for validated targets of miR-103a-3p and miR-378a-5p.

References

1. Chen, W.; Yao, G.; Zhou, K. MiR-103a-2-5p/MiR-30c-1-3p Inhibits the Progression of Prostate Cancer Resistance to Androgen Ablation Therapy via Targeting Androgen Receptor Variant 7. *J. Cell Biochem.* **2019**, *120*, 14055–14064. https://doi.org/10.1002/JCB.28680.
2. Ge, J.; Mao, L.; Xu, W.; Fang, W.; Wang, N.; Ye, D.; Dong, Z.; Guan, H.; Guan, C. MiR-103a-3p Suppresses Cell Proliferation and Invasion by Targeting Tumor Protein D52 in Prostate Cancer. *J. Investig. Surg.* **2021**, *34*, 984–992. https://doi.org/10.1080/08941939.2020.1738602.
3. Yi, Q.; Wei, J.; Li, Y. Effects of MiR-103a-3p Targeted Regulation of TRIM66 Axis on Docetaxel Resistance and Glycolysis in Prostate Cancer Cells. *Front. Genet.* **2022**, *12*, 813793. https://doi.org/10.3389/FGENE.2021.813793/BIBTEX.
4. Singh, P.K.; Preus, L.; Hu, Q.; Yan, L.; Long, M.D.; Morrison, C.D.; Nesline, M.; Johnson, C.S.; Koochekpour, S.; Kohli, M.; et al. Serum MicroRNA Expression Patterns That Predict Early Treatment Failure in Prostate Cancer Patients. *Oncotarget* **2014**, *5*, 824–840. https://doi.org/10.18632/ONCOTARGET.1776.
5. Kooshkaki, O.; Rezaei, Z.; Rahmati, M.; Vahedi, P.; Derakhshani, A.; Brunetti, O.; Baghbanzadeh, A.; Mansoori, B.; Silvestris, N.; Baradaran, B. MiR-144: A New Possible Therapeutic Target and Diagnostic/Prognostic Tool in Cancers. *Int. J. Mol. Sci.* **2020**, *21*, 2578. https://doi.org/10.3390/IJMS21072578.
6. You, B.; Zhang, K.C. MicroRNA-144-3p Inhibits Cell Proliferation and Promotes Apoptosis in Castration-Resistant Prostate Cancer by Targeting CEP55. *Eur. Rev. Med. Pharmacol. Sci.* **2018**, *22*, 7660–7670. https://doi.org/10.26355/EURREV_201811_16383.
7. Sun, X.B.; Chen, Y.W.; Yao, Q.S.; Chen, X.H.; He, M.; Chen, C.B.; Yang, Y.; Gong, X.X.; Huang, L. MicroRNA-144 Suppresses Prostate Cancer Growth and Metastasis by Targeting EZH2. *Technol. Cancer Res. Treat.* **2021**, *20*, 1533033821989817. https://doi.org/10.1177/1533033821989817.
8. Zheng, H.; Guo, Z.; Zheng, X.; Cheng, W.; Huang, X. MicroRNA-144-3p Inhibits Cell Proliferation and Induces Cell Apoptosis in Prostate Cancer by Targeting CEP55. *Am. J. Transl. Res.* **2018**, *10*, 2457.
9. Souza, M.F.; Cólus, I.M.S.; Fonseca, A.S.; Antunes, V.C.; Kumar, D.; Cavalli, L.R. MiR-182-5p Modulates Prostate Cancer Aggressive Phenotypes by Targeting EMT Associated Pathways. *Biomolecules* **2022**, *12*, 187. https://doi.org/10.3390/BIOM12020187/S1.
10. Wang, D.; Lu, G.; Shao, Y.; Xu, D. MiR-182 Promotes Prostate Cancer Progression through Activating Wnt/β-Catenin Signal Pathway. *Biomed. Pharmacother.* **2018**, *99*, 334–339. https://doi.org/10.1016/J.BIOPHA.2018.01.082.
11. Stafford, M.Y.C.; McKenna, D.J. MiR-182 Is Upregulated in Prostate Cancer and Contributes to Tumor Progression by Targeting MITF. *Int. J. Mol. Sci.* **2023**, *24*, 1824. https://doi.org/10.3390/IJMS24031824.
12. Sur, S.; Steele, R.; Shi, X.; Ray, R.B. MiRNA-29b Inhibits Prostate Tumor Growth and Induces Apoptosis by Increasing Bim Expression. *Cells* **2019**, *8*, 1455. https://doi.org/10.3390/CELLS8111455.
13. Worst, T.S.; Previti, C.; Nitschke, K.; Diessl, N.; Gross, J.C.; Hoffmann, L.; Frey, L.; Thomas, V.; Kahlert, C.; Bieback, K.; et al. MiR-10a-5p and MiR-29b-3p as Extracellular Vesicle-Associated Prostate Cancer Detection Markers. *Cancers* **2019**, *12*, 43. https://doi.org/10.3390/CANCERS12010043.

14. Yao, B.; Zhu, S.; Wei, X.; Chen, M.K.; Feng, Y.; Li, Z.; Xu, X.; Zhang, Y.; Wang, Y.; Zhou, J.; et al. The CircSPON2/MiR-331-3p Axis Regulates PRMT5, an Epigenetic Regulator of CAMK2N1 Transcription and Prostate Cancer Progression. *Mol. Cancer* **2022**, *21*, 119. https://doi.org/10.1186/S12943-022-01598-6/FIGURES/10.
15. Oh-Hohenhorst, S.J.; Lange, T. Role of Metastasis-Related MicroRNAs in Prostate Cancer Progression and Treatment. *Cancers* **2021**, *13*, 4492. https://doi.org/10.3390/CANCERS13174492.
16. Karatas, O.F.; Wang, J.; Shao, L.; Ozen, M.; Zhang, Y.; Creighton, C.J.; Ittmann, M. MiR-33a Is a Tumor Suppressor MicroRNA That Is Decreased in Prostate Cancer. *Oncotarget* **2017**, *8*, 60243. https://doi.org/10.18632/ONCOTARGET.19521.
17. Cochetti, G.; Poli, G.; Guelfi, G.; Boni, A.; Egidi, M.G.; Mearini, E. Different Levels of Serum MicroRNAs in Prostate Cancer and Benign Prostatic Hyperplasia: Evaluation of Potential Diagnostic and Prognostic Role. *OncoTargets Ther.* **2016**, *9*, 7545–7553. https://doi.org/10.2147/OTT.S119027.
18. Lin, Y.; Qi, X.; Chen, J.; Shen, B. Multivariate Competing Endogenous RNA Network Characterization for Cancer MicroRNA Biomarker Discovery: A Novel Bioinformatics Model with Application to Prostate Cancer Metastasis. *Precis. Clin. Med.* **2022**, *5*, 1. https://doi.org/10.1093/PCMEDI/PBAC001.
19. Bhagirath, D.; Liston, M.; Patel, N.; Akoto, T.; Lui, B.; Yang, T.L.; To, D.M.; Majid, S.; Dahiya, R.; Tabatabai, L.; et al. MicroRNA Determinants of Neuroendocrine Differentiation in Metastatic Castration-Resistant Prostate Cancer. *Oncogene* **2020**, *39*, 7209–7223. https://doi.org/10.1038/s41388-020-01493-8.
20. Yu, K.J.; Ji, D.Y.; Hsieh, M.L.; Chuang, C.K.; Pang, S.T.; Weng, W.H. EPA Modulates KLK Genes via MiR-378: A Potential Therapy in Prostate Cancer. *Cancers* **2022**, *14*, 2813. https://doi.org/10.3390/CANCERS14112813.
21. Cannistraci, A.; Hascoet, P.; Ali, A.; Mundra, P.; Clarke, N.W.; Pavet, V.; Marais, R. MiR-378a Inhibits Glucose Metabolism by Suppressing GLUT1 in Prostate Cancer. *Oncogene* **2022**, *41*, 1445–1455. https://doi.org/10.1038/s41388-022-02178-0.
22. Chen, Q.G.; Zhou, W.; Han, T.; Du, S.Q.; Li, Z.H.; Zhang, Z.; Shan, G.Y.; Kong, C.Z. MiR-378 Suppresses Prostate Cancer Cell Growth through Downregulation of MAPK1 In Vitro and In Vivo. *Tumor Biol.* **2016**, *37*, 2095–2103. https://doi.org/10.1007/S13277-015-3996-8/METRICS.
23. Mao, Y.; Li, W.; Hua, B.; Gu, X.; Pan, W.; Chen, Q.; Xu, B.; Lu, C.; Wang, Z. Circular RNA_PDHX Promotes the Proliferation and Invasion of Prostate Cancer by Sponging MiR-378a-3p. *Front. Cell Dev. Biol.* **2021**, *8*, 602707. https://doi.org/10.3389/FCELL.2020.602707/BIBTEX.
24. Avgeris, M.; Stravodimos, K.; Scorilas, A. Loss of MiR-378 in Prostate Cancer, a Common Regulator of KLK2 and KLK4, Correlates with Aggressive Disease Phenotype and Predicts the Short-Term Relapse of the Patients. *Biol. Chem.* **2014**, *395*, 1095–1104. https://doi.org/10.1515/HSZ-2014-0150/DOWNLOADASSET/SUPPL/HSZ-2014-0150.ZIP.
25. Blondal, T.; Jensby Nielsen, S.; Baker, A.; Andreasen, D.; Mouritzen, P.; Wrang Teilum, M.; Dahlsveen, I.K. Assessing Sample and MiRNA Profile Quality in Serum and Plasma or Other Biofluids. *Methods* **2013**, *59*, S1–S6. https://doi.org/10.1016/J.YMETH.2012.09.015.
